# Preliminary User Evaluation of a New Dental Technology Virtual Simulation System: Development and Validation Study

**DOI:** 10.2196/36079

**Published:** 2022-09-12

**Authors:** Mengwei Pang, Xiaohan Zhao, Daiyu Lu, Yihan Dong, Lin Jiang, Jie Li, Ping Ji

**Affiliations:** 1 College of Stomatology, Chongqing Medical University Chongqing China; 2 Chongqing Key Laboratory of Oral Diseases and Biomedical Sciences Chongqing China; 3 Chongqing Municipal Key Laboratory of Oral Biomedical Engineering of Higher Education Chongqing China; 4 Stomatological Hospital of Chongqing Medical University Chongqing China; 5 State Key Laboratory of Virtual Reality Technology and Systems, Beihang University Beijing China

**Keywords:** virtual simulation, dental technology, OSCE, virtual reality, dentistry, dental technician, framework, certified dental technician, development, validation, serious game, dental technology, dental, technology

## Abstract

**Background:**

With the advancements in the dental health care industry, the demand for dental technicians has increased. Dental technicians should be thoroughly assessed and trained in practical skills and pass professional certification examinations to ensure that they are competent to work closely with dentists. Unfortunately, such training courses and tests are in short supply worldwide. The use of virtual simulation technology can help solve these problems.

**Objective:**

This study presents a new strategic framework design for a certified dental technician practical examination called as the certified Objective Manipulative Skill Examination of Dental Technicians (OMEDT), which is based on the Objective Structured Clinical Examination (OSCE). We present the development and validation of the OMEDT system, a new virtual simulated training system, to meet the demands of the OMEDT framework. The combination of OMEDT and the OMEDT system can solve the complex problems encountered in the certified dental technician practical examination with excellent efficiency, high quality, and low cost.

**Methods:**

The OMEDT framework design was constructed according to the OSCE guide and the Chinese vocational skill standards for dental technicians. To develop the OMEDT system, we organized a new framework based on the virtual learning network platform, the haptic feedback system, and the real-time dental training and evaluation system. The effectiveness evaluation of the OMEDT system was divided into 2 phases: in the first phase, 36 students were recruited to use the test module to finish the task and their performance data were collected and analyzed; and in the second phase, a questionnaire was administered to 30 students who used the system for their studies and graduation exams.

**Results:**

The OMEDT and the corresponding skill training virtual simulation OMEDT system were developed, and preliminary user evaluation was performed to assess their effectiveness and usefulness. The OMEDT system was found to improve students’ practical skills by training with the evaluation results. In addition, several key research topics were explored, including the effects of positive feedback of the knowledge of results on the improvement of the students’ skill level and the common sense transformation of educators in the virtual simulation technology environment.

**Conclusions:**

The development of OMEDT and the OMEDT system has been completed and their effectiveness has been verified.

## Introduction

The dental health care industry is a technically oriented discipline [1]. With the advancements in the dental industry, the demand for dental technicians has increased. Dental technology, which has originated from traditional dentistry, has become an independent field of study in China where students are trained in the latest technology of dental prostheses [2]. Skill is critical for the professional development of dental technicians, including the cognitive ability to apply theoretical concepts, professional psychomotor skills in dental technology laboratories, affective skills of communicating with dentists, and the responsibility of maintaining the prostheses quality. The skill level of a dental technician determines the quality of the denture processing, which then affects the success of the treatment. Unfortunately, relevant skill training courses incur high costs, as they require a significant capital investment in equipment and materials and high educator/student ratios to maintain close supervision [3-7]. Moreover, limitations in the development of dental technology are reflected by the lack of a robust certification system in the industry [8,9]. The certified dental technician (CDT) practical examination, which is the vocational assessment of dental technology education, is not conducted worldwide.

In light of the above limitations, the assessment of dental technology skills can be developed following the pattern of the stomatology professional certification practical examination. The Objective Structured Clinical Examination (OSCE) is a widely used strategy for the stomatology professional certification practical examination worldwide. Dental institutes in various countries, including China, use OSCEs for formative and summative assessments of students’ clinical competence [10-12]. OSCE is a tool used for evaluating performance in simulated environments; it consists of a series of standardized assessment stations, requiring students to perform several tasks based on clinical situations. Either standardized or alternative simulators are used. The examinees are assessed using the same stations and rating schemes within the same timeframes, and the examiners assess the examinees’ application of knowledge to practice [13]. Over the past few decades, OSCE has proven to be a valid and reliable tool for assessing all 3 learning domains of cognitive skills, psychomotor skills, and affective skills [11,14]. However, for dental technology students, OSCE is a complex, resource-intensive, and time-intensive assessment examination. Unlike dental clinical skills, skills associated with fabricating corrective devices and replacements require more process time, resources, semifinished products, etc. Thus, it is necessary to embrace new technologies, reset the strategic framework, create more immersive simulation environments, and transform OSCEs at the application level to meet the needs of CDT practice examinations.

Recent research studies [15,16] have found that virtual reality simulation technologies could create more immersive simulation environments and may positively affect dental education. The existing virtual simulation technology is mainly divided into the virtual learning network platform (VLNP), the haptic feedback system, and the real-time dental training and evaluation system (RDTES). VLNP is usually designed as a series of web-based skill training modules. It helps students grasp the operational essentials and improves cognitive ability and affective skills. Compared with virtual reality technology, VLNP is less realistic, but it has the advantage of being less expensive and can be built on serious game theories [15,16]. For developing a multimodule virtual simulation system, VLNP is a more suitable option considering the costs. Inexperienced dental technicians or students may be exposed to occupational hazards and risks, thereby threating public safety. Such hazards can be avoided by using haptic feedback technology to build virtual training environments. Haptic interaction has been successfully applied in dental technology skill training to simulate specific tasks [17]. RDTES is a preclinical simulator that provides real-time image processing using 3D graphics and video recordings. Recent studies [18,19] have shown that if students see illustrations of their procedures, they can understand any inadequacies in their skills objectively and visually. However, another study [20] has shown that RDTES cannot wholly replace the feedback that educators give students during teaching activities. In RDTES technology, the inclusion and recording of educators’ assessments have always been an ongoing problem.

The advantages of virtual simulation technologies include safety, time saving, economic benefits, ethical benefits, increased frequency and relevance of training, and teaching error management [21-38]. The disadvantages include less realistic force feedback simulation, insufficient training content, and unquantified evaluation of the training results [39]. To meet the requirements of the CDT practical examination, many technical and structural issues need to be addressed. Unfortunately, although many attempts have been made to develop a dental simulator by different manufacturers for the psychomotor skills training of dental students, there has never been a system designed explicitly for the professional performance training of dental technology students and the CDT practical examination. Thus, this study presents a new strategic framework design for the CDT practical examination based on OSCE, called as the certified Objective Manipulative Skill Examination of Dental Technicians (OMEDT). Then, we present the development and validation of a new psychomotor skill training virtual simulation system, the OMEDT system, to meet the OMEDT framework’s demands. The OMEDT system is based on the VLNP–haptic feedback–RDTES (V-H-R) architecture, specifically for dental technology students and the CDT practical examination. Experiments were designed to evaluate the effectiveness of this system. The purpose of this study was to develop a new examination framework and the corresponding skill training virtual simulation system for dental technology students, evaluate the effect of the trainings through virtual simulation on dental technology students’ professional performance during preclinical laboratory work, and discuss the possibility of utilizing virtual simulation technology in the CDT practical examination.

## Methods

### Framework Design of OMEDT

The OMEDT framework design was constructed in strict accordance with the OSCE:AMEE Guide [11,12] and the national vocational skill standards for dental technicians [40], which were presented by the National Health Commission of the People’s Republic of China. Based on the talent cultivation mode oriented by industry demand, most universities follow this standard in designing professional performance training strategies and curricula for their dental technology majors. This approach ensures that most universities’ dental technology graduates have the ability to pass the OMEDT and that graduates who pass the OMEDT are prepared to work in the industry.

For critical technologies with common characteristics in different technological processes, the design steps in this strategy have been deleted, combined, and supplemented. Moreover, as per the time taken to complete the technical processes, the examination is divided into short-term completion projects and long-term completion projects and the individual combination of the examination stations is determined by random draws. Based on the development concept of “combining virtual with the actual environment,” the OMEDT system must be used to complete the virtual simulation site in the OMEDT.

### Design and Compilation of the OMEDT System

The OMEDT system was developed considering the following requirements:

1. Increase the opportunities of dental technology students’ professional psychomotor skills training.
2. Turn complex experiments into serious games to gradually acquire new problem-solving skills and domain knowledge.
3. Decrease the requirement of dental laboratory equipment and the waste of experimental materials due to inexperienced psychomotor skills.
4. Spend more time learning skills and less time waiting in line for faculty feedback, shortening the amount of time spent in the dental laboratory.
5. Establish an instructor–student technician communication mechanism that provides immediate feedback.
6. Analyze and store students’ training data and instructors’ comment data.
7. Cover the major process of dental technology’s curriculum and satisfy the demand of the CDT practical examination.

Based on the above considerations, the OMEDT system selected the following contents for module development in the first phase of the study: (1) prosthesis casting process simulation, (2) tooth morphology sketch design simulation, (3) removable partial denture (RPD) design (D-RPD) simulation, (4) esthetic dental photography and design simulation, (5) dies diagnostic simulation, and (6) tooth engraving haptic feedback simulation. The structure of the data management module is designed to manage the above contents. The OMEDT system was developed with the Unity engine (Unity Technologies, Unity Pro) and the C++11 and C# 4.0 developing languages. The teeth data were collected by computed tomography and intraoral 3D scanning. AutoCAD 3ds Max was used to refine the shape of the geometric models. To achieve vivid physical simulation, the physical skeleton of the geometric environment was constructed. The gypsum blocks used for carving were modeled as triangle meshes. With the 6 degrees of freedom (6-DOF) haptic rendering software development toolkit provided by the State Key Laboratory of Virtual Reality Technology and Systems of Beihang University, we succeeded in simulating the operation process of tooth carving with force feedback [41,42].

### Performance Evaluation of the OMEDT System

The performance evaluation of the OMEDT system was divided into 2 phases. Phase I consisted of user tests for specific modules. Phase II consisted of a questionnaire survey for long-term users.

#### Phase I

The OMEDT was initially designed as a whole life cycle training framework for promoting students’ professional performance. It consists of many different modules, including casting and D-RPD. A single student should undertake specific modules to train according to his or her learning progress. For the construction validity evaluation of the OMEDT system, the V-H-R architecture and the learning structure of students should be considered simultaneously. Test modules with high coverage in VLNP and RDTES dimensions should be selected to perform experimental evaluation. Therefore, the D-RPD module was selected to perform the evaluation experiment. D-RPD is an important core content in dental technology majors, and the courses related to this content will last at least 3 semesters in the training plan, with wide coverage in the learning period. The data volume and data dimension in the D-RPD module are relatively full, which is a great challenge for the execution of the data management module. Experiments using this module can check the execution effect of VLNP and RDTES in the V-H-R architecture at the same time. D-RPD is the most difficult core course for students of stomatology technology to fully master because of its complex content and the need for many instructors with extensive experience in D-RPD. The construction validity of the OMEDT can be fully illustrated by the verification of the difficulty levels in the modules.

In the first phase of the short-term system usability tests, 36 students majoring in dental technology were recruited to participate in the experiments, and they signed informed consent forms. To ensure the consistent level of expertise, all participants were from the same class at the College of Stomatology, Chongqing Medical University and received the same level of instruction in D-RPD theory. Before formal evaluation experiments, every participant received sufficient pretraining to become familiar with the OMEDT system and the testing module, including the use of computer equipment, software-related operations, etc. The completion of the pretraining is based on students being able to draw RPD designs by using various tools within the D-RPD module. Each student took 7 weeks to complete the formal evaluation experiments. The students were required to complete and submit the design of RPDs based on actual clinical cases with a D-RPD module per week. Students used their own personal computers to complete tasks in different network environments. These tasks were designed as additional assignments for the course. Students were told that completing these tasks would not give them extra points but would help them improve their design skills. Each design was scored by 2 experienced dental technology instructors with the testing module. The experiment was monitored by the OMEDT system developer team, which consisted of 3 technical support engineers and 1 dental technology instructor to deal with any potential system bugs. Data were collected from 2 dimensions: task completion time and task score. The D-Manager module was used to register and extract the original data and download it as a table. The data were analyzed using GraphPad Prism 8.0.

#### Phase II

In the second phase, 1 questionnaire was administered to 30 dental technology students who used the OMEDT system for a 1-year study and graduate examination. The questionnaire was designed to collect user feedback on using the OMEDT system, including the system’s efficiency improvement for learning and working, improvement of skills and theoretical knowledge, improvement of independent learning ability, and acceptance of using the system for examinations. This preliminary questionnaire was piloted using 10 students who did not participate in the main study to ensure the clarity of the questionnaire. On the basis of the feedback, a panel discussion was organized by 2 senior instructors to revise some ambiguous words that might cause misunderstanding, avoid 2-in-1 questions, and make the questions more concrete. Finally, 9 items were ascertained, among which the participants rated 8 items by using a 5-point Likert scale. An open-ended question was used to collect suggestions for the future development and limitations of the OMEDT systems in dental technology education. The data were analyzed using GraphPad Prism 8.0.

### Ethics Approval

The research ethics committee of the Affiliated Hospital of Stomatology, Chongqing Medical University approved this study (COHS-REC-2022 [LSNo 080]).

## Results

### OMEDT Framework

The OMEDT is organized as a 1-day examination, divided into a half-day short assessment and a half-day long assessment. The short assessment consists of 4 compulsory stations and 2 random stations. The 4 compulsory stations are set to take 15 minutes to complete, the 2 random stations are set to take 45 minutes to complete, and the candidate transfer time between the stations is considered as 5 minutes. The long assessment consists of 1 random test station, which is set to take 3 hours to complete. Since the total time taken to complete the short and long assessments are almost the same and the time in each station in the short assessment is fixed, the candidates need to randomly draw lots before the OMEDT begins to determine their test station combination and test sequence for long and short assessments.

### OMEDT System

The OMEDT system supports high-quality real-time 3D interaction. Multiple art resources are built into the system, including pictures, 3D models, and animations. To guarantee the rendering quality, the number of polygon meshes of a single scene can be up to 10 million polygons, and the resolution is less than 0.01 mm. In addition, the update frequency can be as high as 120 per frame. Bezier curve drawing, spline curve drawing, and other drawing tools are supported in the OMEDT system; the minimum error of drawing is not higher than 0.01 mm. The tooth-carving part of the OMEDT system is based on the Unidental haptic-based dental simulator 6-DOF haptic rendering, and the simulation of bimanual haptic feedback is provided for the students to practice hand, eye, and foot coordination. Based on the V-H-R architecture, the OMEDT system has 7 main modules: D-Casting, D-Sketching, D-RPD, D-Smile, D-Dies, D-Carving, and D-Manager. These modules are for prosthesis casting (D-Casting), dental sketching (D-Sketching), D-RPD, esthetic dental photography and design (D-Smile), dies diagnosis (D-Dies), tooth carving haptic feedback (D-Carving), and integrated management (D-Manager). These are standardized into 4 main professional performance training design layouts:

1. Process simulation based on the demand of dental laboratory technology in reality and the regularization theory framework of the serious game carried out on the VLNP (D-Casting).
2. Design simulation based on the clinical cases and the blended learning instantaneously, carried out on the VLNP (D-Sketching, D-RPD, D-Smile).
3. Diagnosis simulation based on the clinical prescription and the modular assembly random database, carried out on the VLNP (D-Dies).
4. Haptic feedback simulation based on hand-eye-foot coordination and the assessment of multiple dimensions, carried out on the Unidental haptic device (D-Carving).

**Figure 1:**
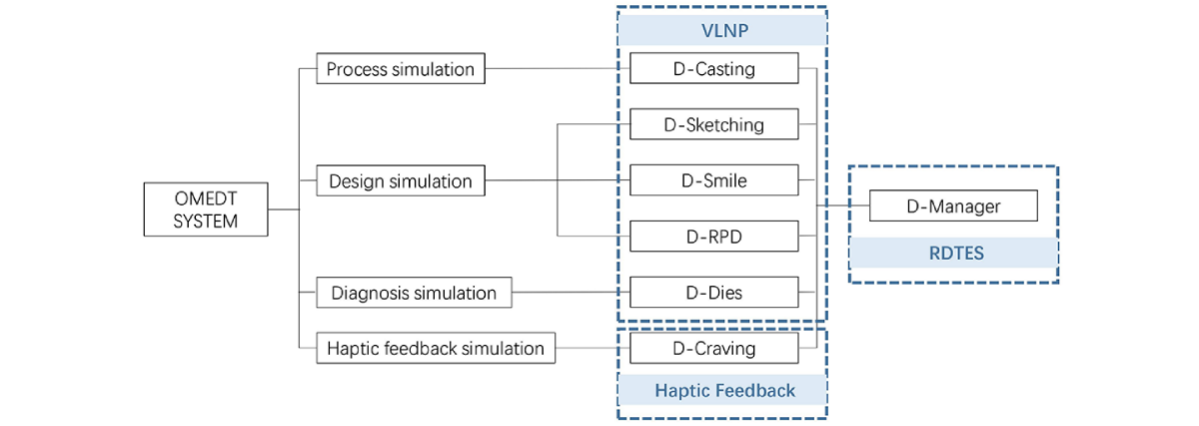
Architectural design of the Objective Manipulative Skill Examination of Dental Technicians system. D-RPD: removable partial denture design; OMEDT: Objective Manipulative Skill Examination of Dental Technicians; RDTES: real-time dental training and evaluation system; VLNP: virtual learning network platform.

**Figure 2:**
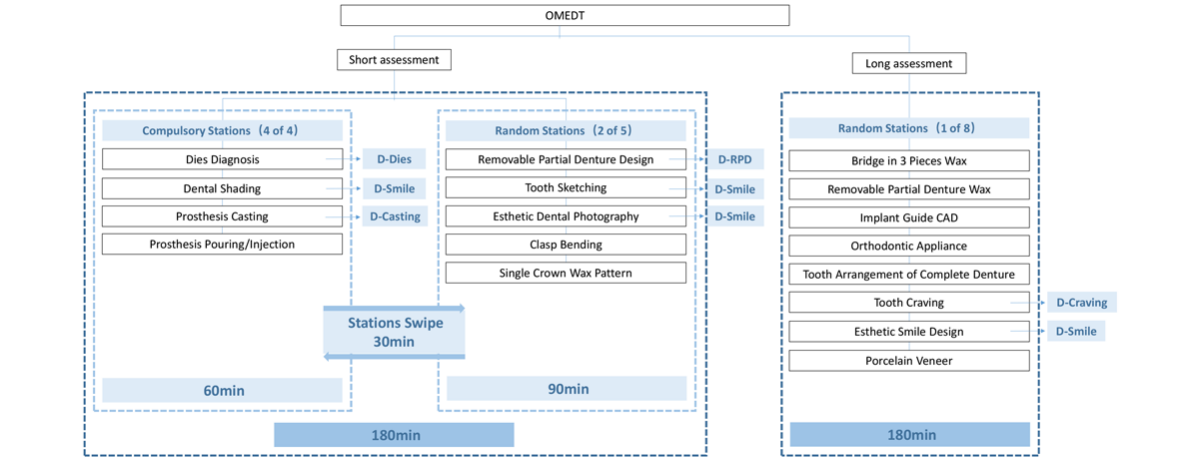
Architectural design of the Objective Manipulative Skill Examination of Dental Technicians and the corresponding modules in the Objective Manipulative Skill Examination of Dental Technicians system. CAD: computer-aided design; OMEDT: Objective Manipulative Skill Examination of Dental Technicians.

**Figure 3:**
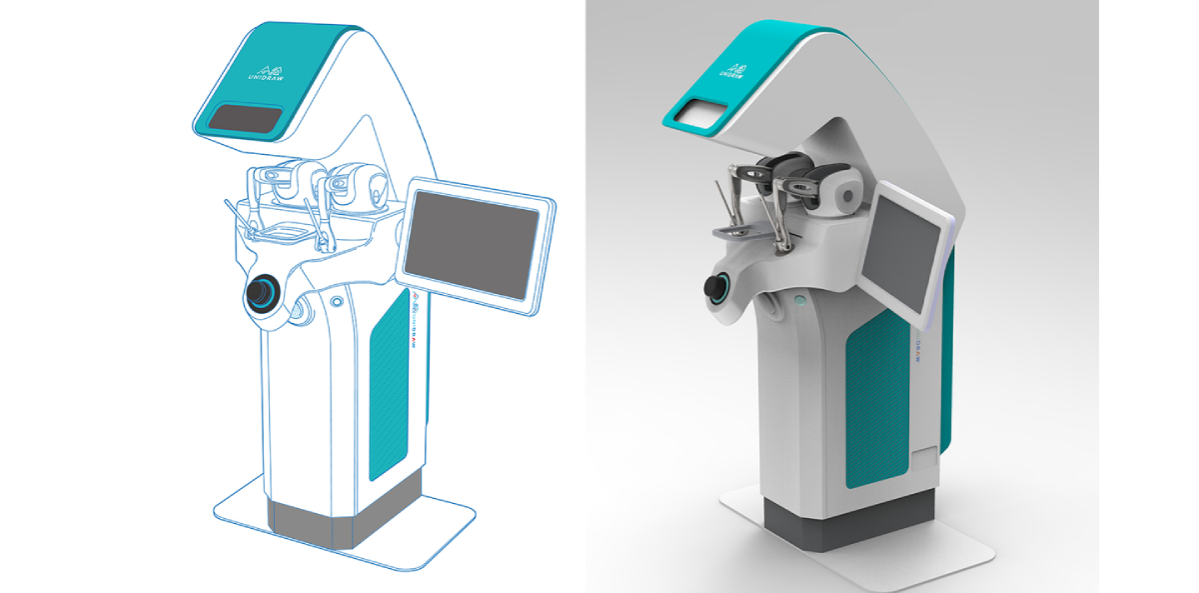
Unidental haptic feedback device mounted on the D-Carving module.

An integrated management system (D-Manager) based on data recording/integration/analysis was used to manage 6 modules. It can assist instructors in completing lecture teaching/homework review/objective structured examination design/test release and other functions while supporting students to practice freely and carry on the RDTES. The VLNP and RDTES modules are recommended to be accessed using the latest version of Google Chrome. The haptic feedback module was developed by Unidental, a third-generation dental simulator developed by the Beijing Unidraw Virtual Reality Technology Research Institute Co Ltd (Figures 1-3).

#### D-Casting

**Figure 4:**
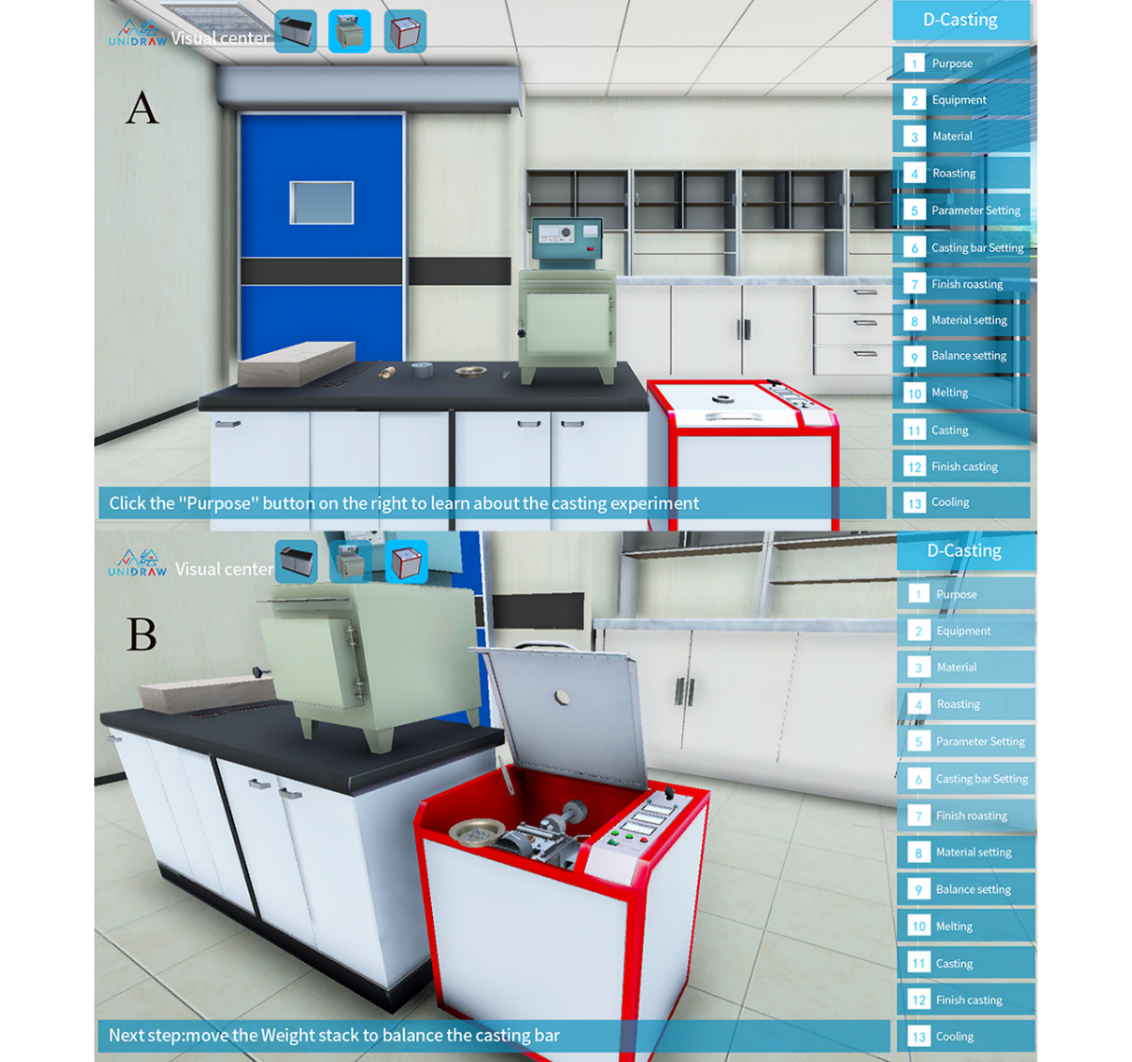
Application programming interface of D-Casting. A: Panorama of the virtual casting laboratory in D-Casting; B: Prosthesis casting process simulation in D-Casting.

Unlike other virtual simulation systems or modules, D-Casting’s approach to design follows the theoretical framework of serious games. In addition, the fabrication of a complete reward/punishment mechanism was designed to increase interest in the operation, and game technology is used to demonstrate those processes that could not be observed in reality, which leads to intense and passionate involvement, goals that motivate, and rules that provide structure. More importantly, D-Casting makes it possible to include the prosthesis casting process in the CDT practical examination, because lengthy processes can be speeded up in simulation, unaffected by semifinished products or any impact of human interference, thereby eliminating the potential for fraud and ethical issues (Figure 4).

#### D-Sketching

**Figure 5:**
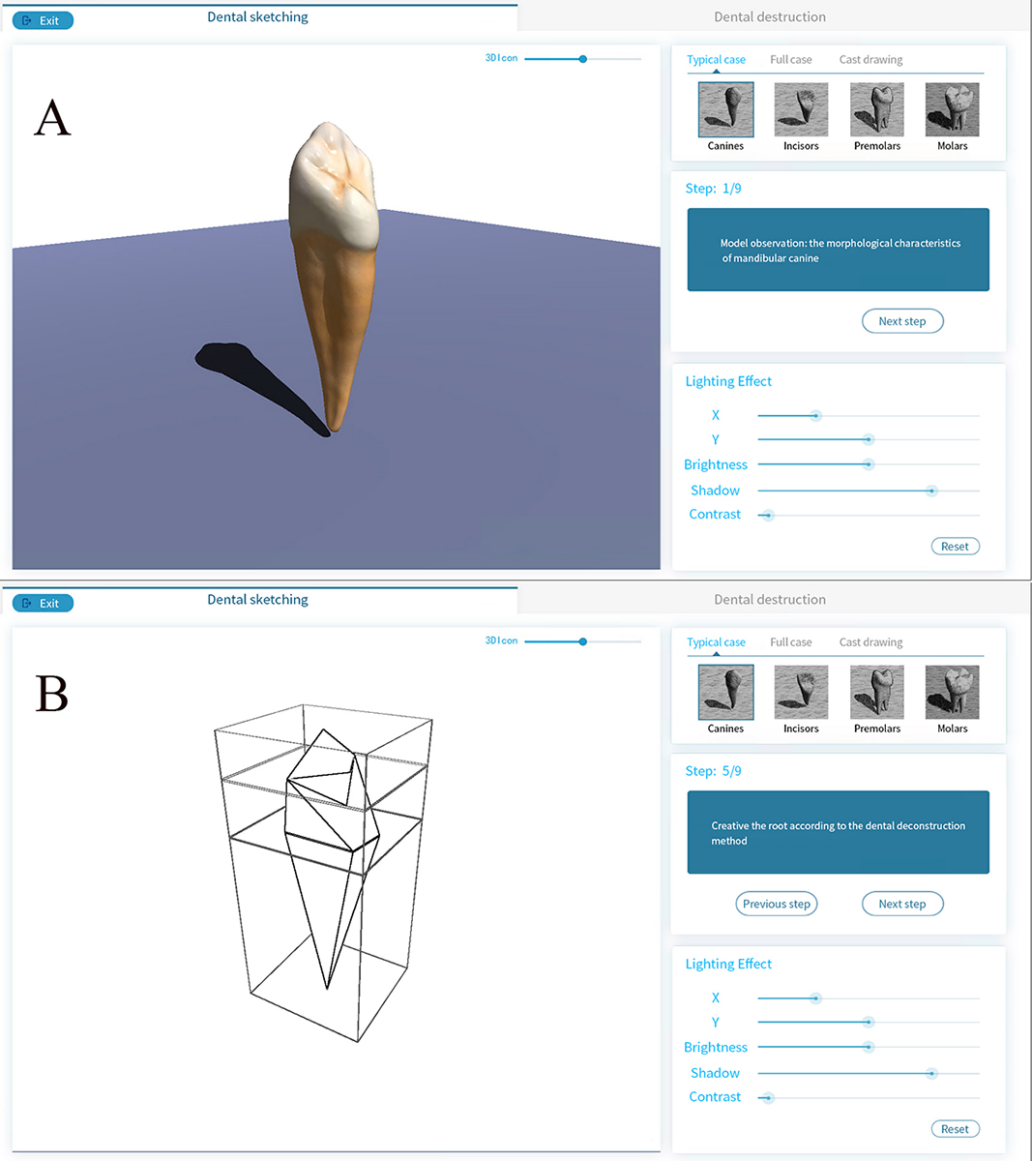
Application programming interface of D-Sketching. A: Panorama of the real-time rendering engine in D-Sketching; B: Sketching process based on the dental deconstruction method.

Combined with an ingenious dental deconstruction method, D-Sketching provides a new approach to learning dental morphology sketches for inadequately trained dental technology students in the art area. The real-time rendering engine fabricates a movable virtual platform for students. They can observe the tooth with the simulation of the light, analyze the light and dark tones on teeth, and perform omnidirectional exercises on sketching to improve their skills. The “tooth assembly mode,” based on dental deconstruction, is provided in D-Sketching. Students could use this module to finish the sketch without actual models based on mastering the morphology of the teeth. D-Sketching could help students build kinesthetic memories from sketching, review the dental anatomy from variable directions after rotation, and reconstruct by sketching. This process also conforms to Fitts and Posner’s seminal model, the most well-used and well-recognized theory of psychomotor skill learning with a long history of use in health care [43] (Figure 5).

#### D-RPD

**Figure 6:**
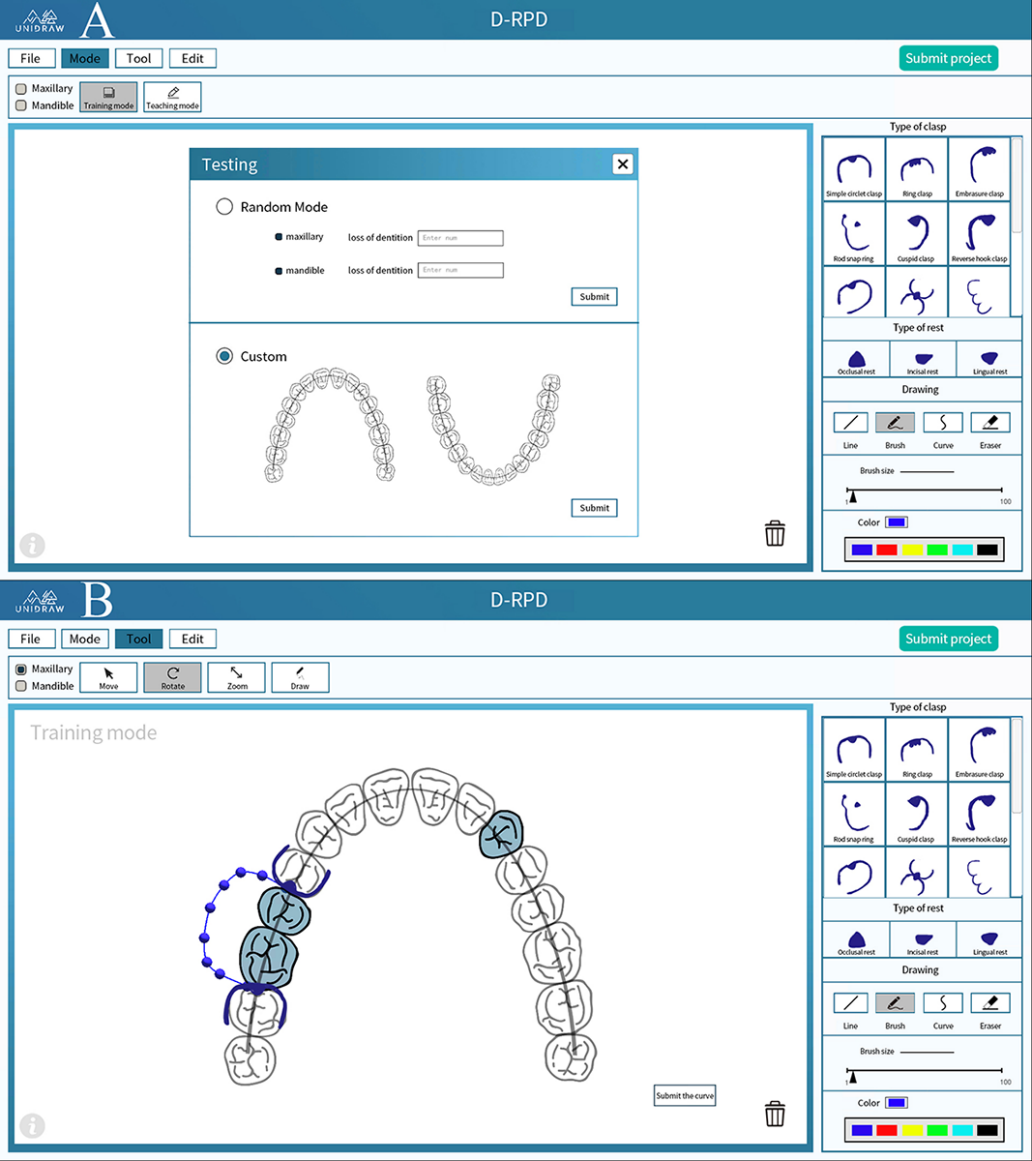
Application programming interface of the removable partial denture design. A: Free practice mode in the removable partial denture design module; B: Random testing mode in the removable partial denture design module.

Based on the clinical case database, D-RPD provides overall solutions for students and instructors. The traditional mode of written D-RPD instructions was abandoned. The virtual dies reconstructed by the scanner were integrated into the database. After students observed the virtual model in D-RPD, they used D-RPD to fabricate electronic instructions. Instructors use D-RPD to review electronic instructions, and scores and feedback can be given in a timely manner. D-RPD supports students in performing independent exercises anytime and anywhere and provides the possibility for large-scale D-RPD examinations (Figure 6).

#### D-Smile

**Figure 7:**
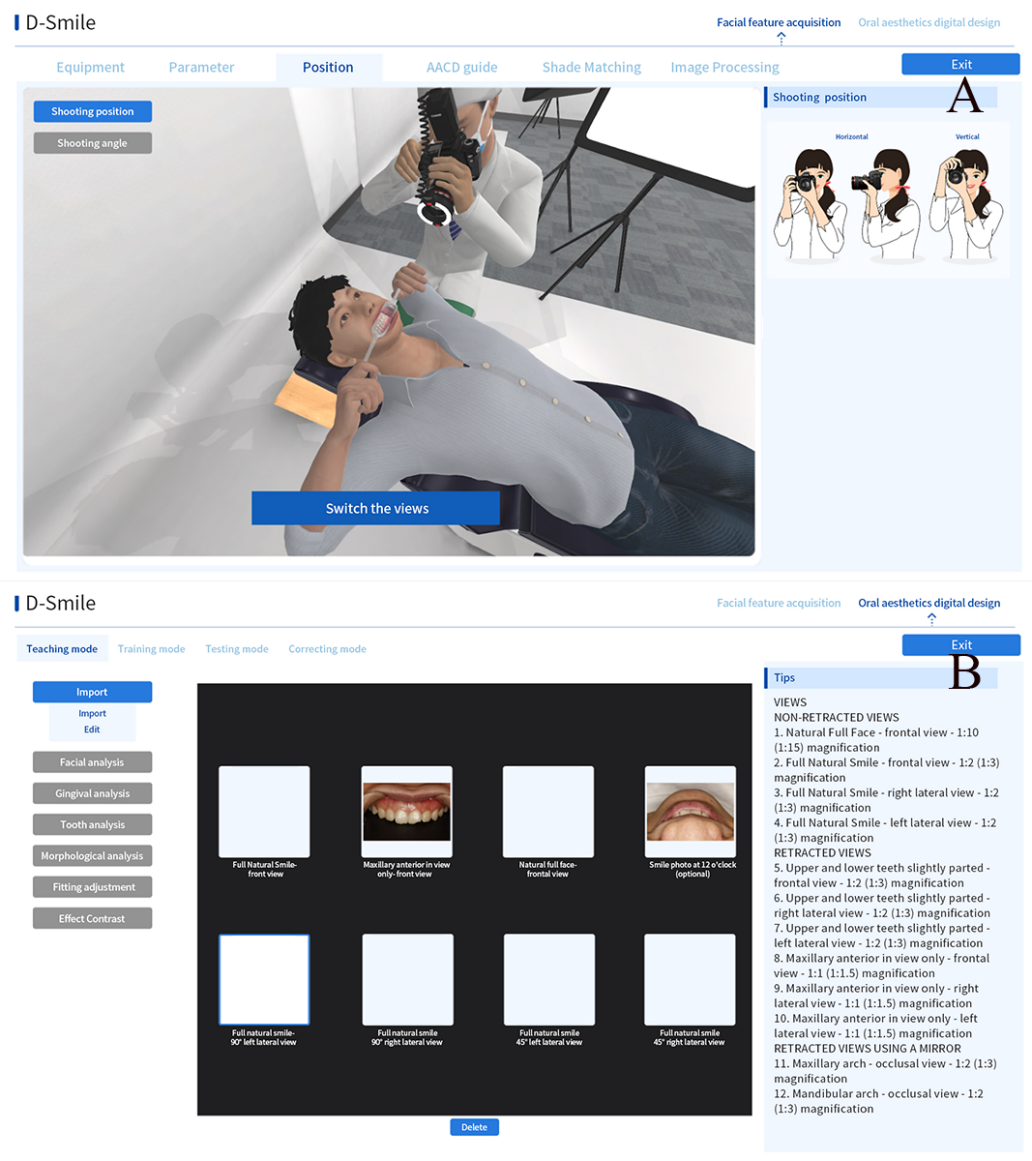
Application programming interface of D-Smile. A: Panorama of a virtual oral photography studio in D-Smile; B: Oral digital aesthetic design based on facial features (referring to the accredited photography guide of the American Academy of Cosmetic Dentistry).

As per the American Academy of Cosmetic Dentistry [44], the whole process of dental macrophotography was simulated in D-Smile. The digital single lens reflex camera and the dental photography studio were disassembled in the simulation, and students could learn dental aesthetic photography techniques online without buying expensive equipment. Based on the teaching objectives, preoperative aesthetic design parts and clinical case databases were implanted into D-Smile. Similar to D-RPD, D-Smile supports students in performing independent exercises anytime and anywhere and provides the possibility for large-scale preoperative aesthetic design examinations (Figure 7).

#### D-Dies

**Figure 8:**
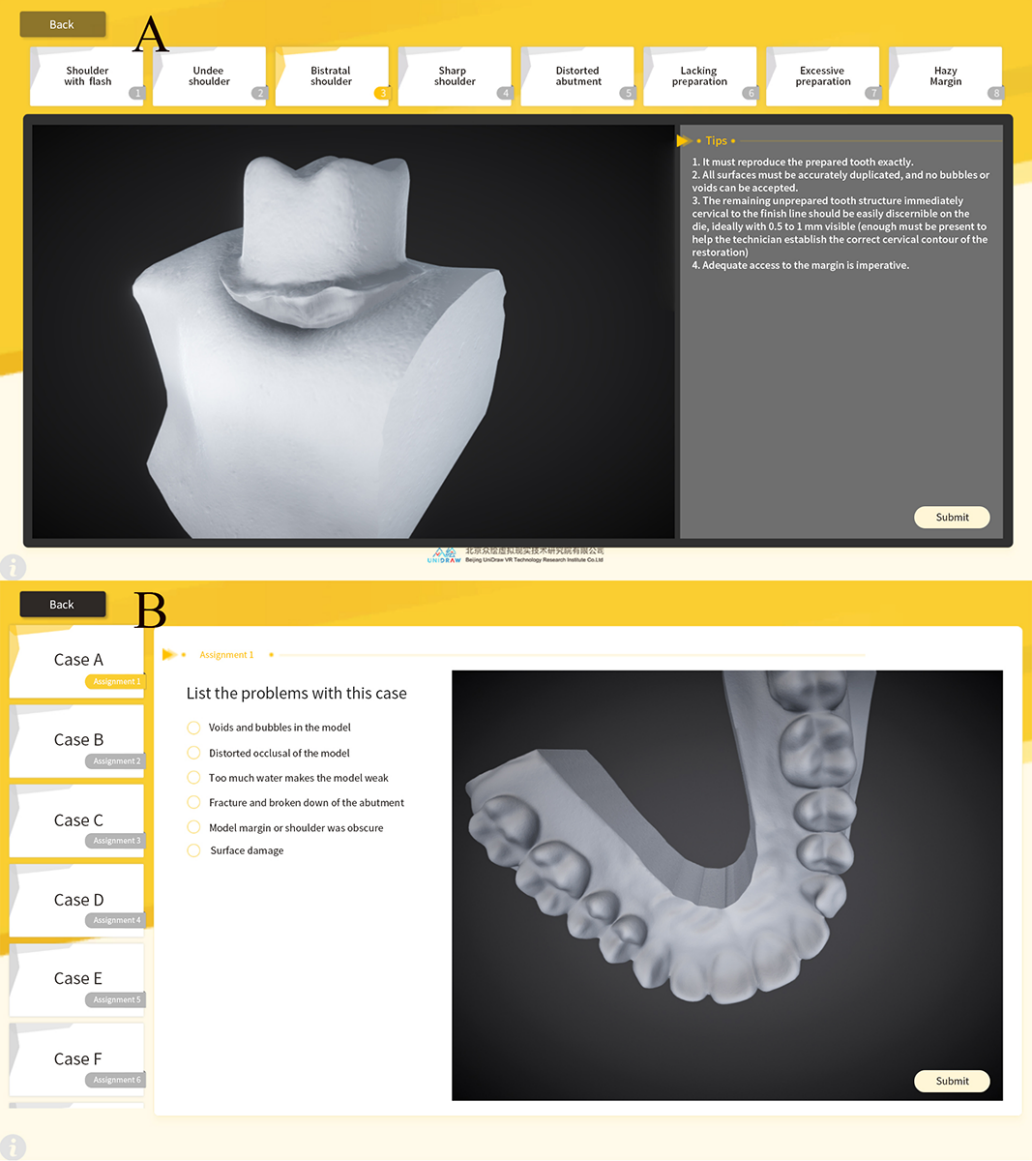
Application programming interface of D-Dies. A: The virtual defect dies segmented by modularization in D-Dies; B: Randomly reconstructed virtual defect dies testing in D-Dies.

When the patient’s intraoral gypsum dies enter the dental laboratory workflow, they usually need to be diagnosed by experienced dental technicians first, to ensure that the accuracy of the gypsum dies meets the production requirements, which also is the basis of the cognitive skills of dental technology students. Defective virtual models are classified strictly by defect types and randomly assembled in the database according to the logical framework of the modular assembly. In D-Dies, the defect dies reconstructed haphazardly will be diagnosed by the students, and then an electronic specification will be formed according to the suggestions. Instructors use D-Dies to review the electronic instructions, and scores and feedback can be given in a timely manner. Likewise, D-Dies supports students in performing independent exercises anytime and anywhere and provides the possibility for large-scale examinations (Figure 8).

#### D-Carving

**Figure 9:**
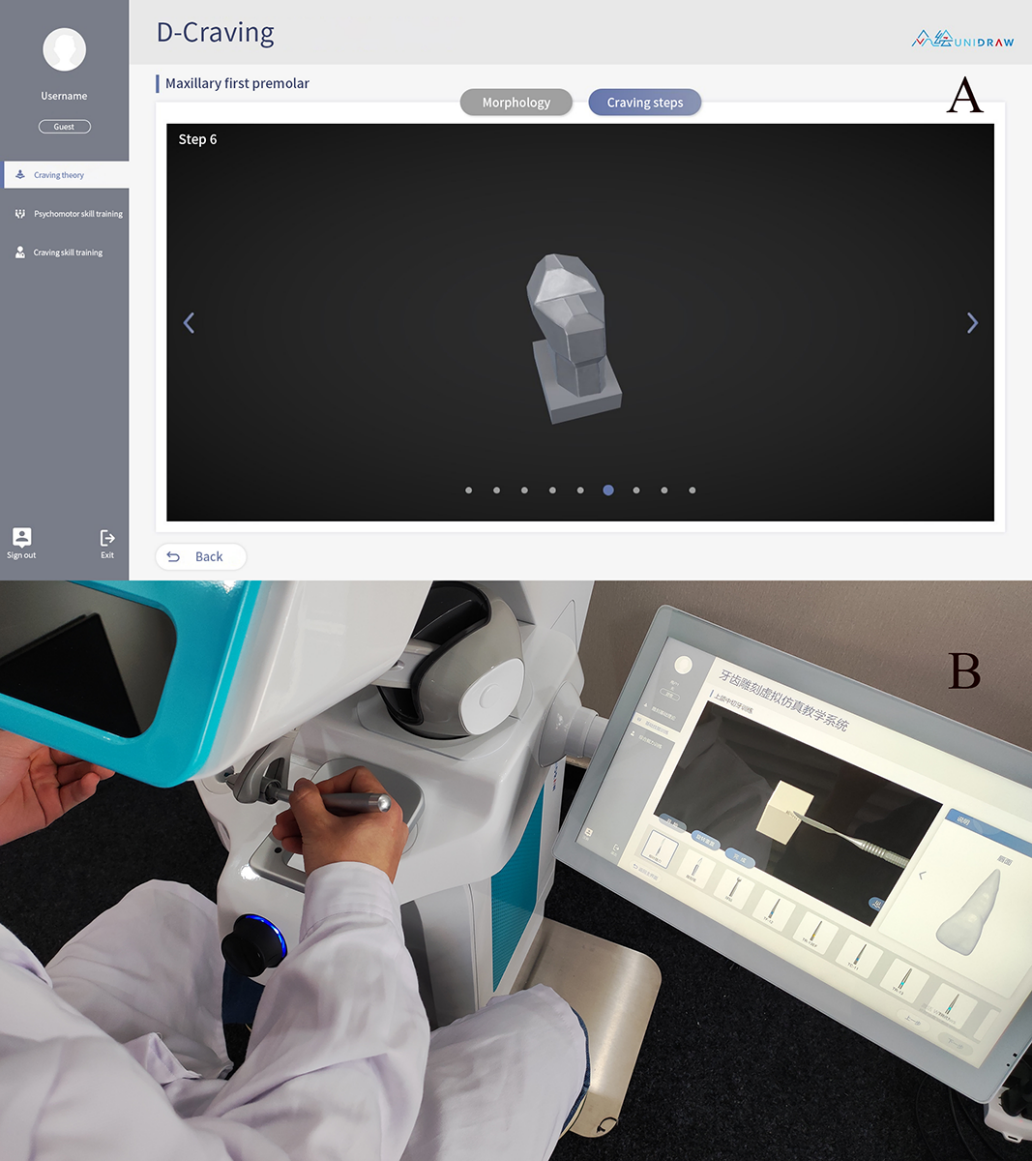
Application programming interface of D-Carving. A: Panorama of the virtual carving steps in D-Carving; B: A student using the D-Carving module to practice tooth carving.

Owing to the tedious and complicated process of tooth carving and the limitations of labor and time, material cost, and safety, students have few opportunities to practice, and it is difficult for them to develop psychomotor skills. D-Carving is the first tooth carving training haptic system in the world. It uses a 6-DOF haptic rendering algorithm based on configuration optimization [42] to form stable multipoint interactions between the carving tool and the carving material within 1 millisecond. The system is equipped with different virtual tools such as dental ball drills, diamond needles, carving pens, and willow carving knives. D-Carving can simulate the texture of wax blocks, plaster, and other materials. The method of marching cube rendering based on a directed distance field and graphics processing unit acceleration can accurately simulate the dynamic change of the topological structure in material removal. The rendering accuracy reaches 0.1 mm to meet the training needs of fine-tooth carving. During the training, the standard control group can be added for comparison, and the evaluation module can be added (Figure 9).

#### D-Manager

D-Manager can publish tests and assessments for any module in the background and support the instructor in marking homework online. Multiple dimensions of training data were collected, including students’ training time in modules, students’ work files, scores given by various instructors to the same student, etc. All data are stored and analyzed on the server to form charts. Instructors and students can master the training progress and adjust the training frequency and training time by checking the chart’s data. Some VLNP parts of the OMEDT system can be used in iLab-x, which is the largest virtual simulation experiment online teaching center in China [45].

### Experimental Results of System Effectiveness

#### Phase I

**Figure 10:**
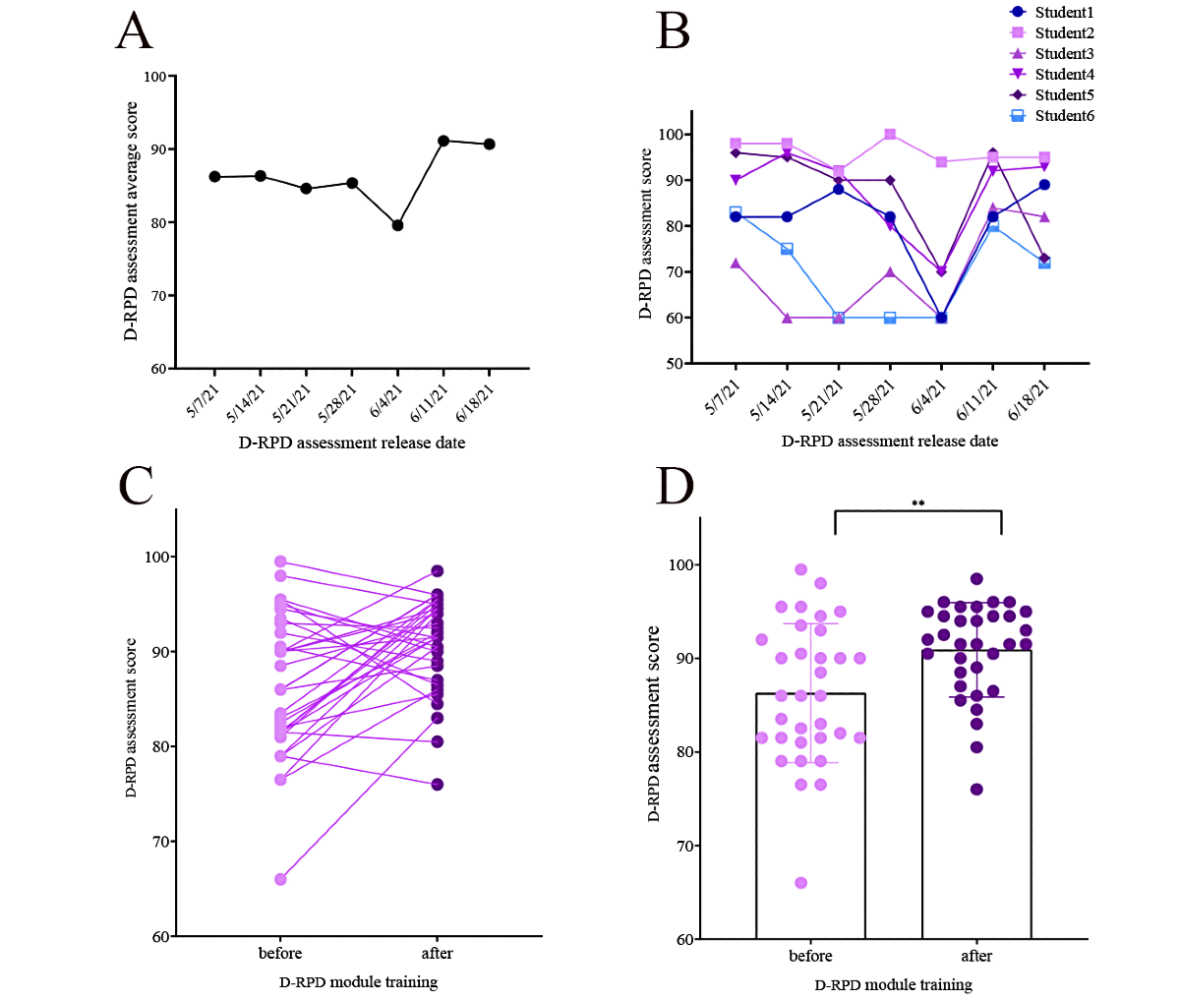
Experimental results of the removable partial denture design (score dimension). A: Average time consumption of the removable partial denture design assessment (33 students in 7 weeks); B: Removable partial denture design assessment score of a sample team (6 students in 7 weeks); C and D: Paired *t* test analysis results of the score-dimension data set, *P*=.002. D-RPD: removable partial denture design.

**Figure 11:**
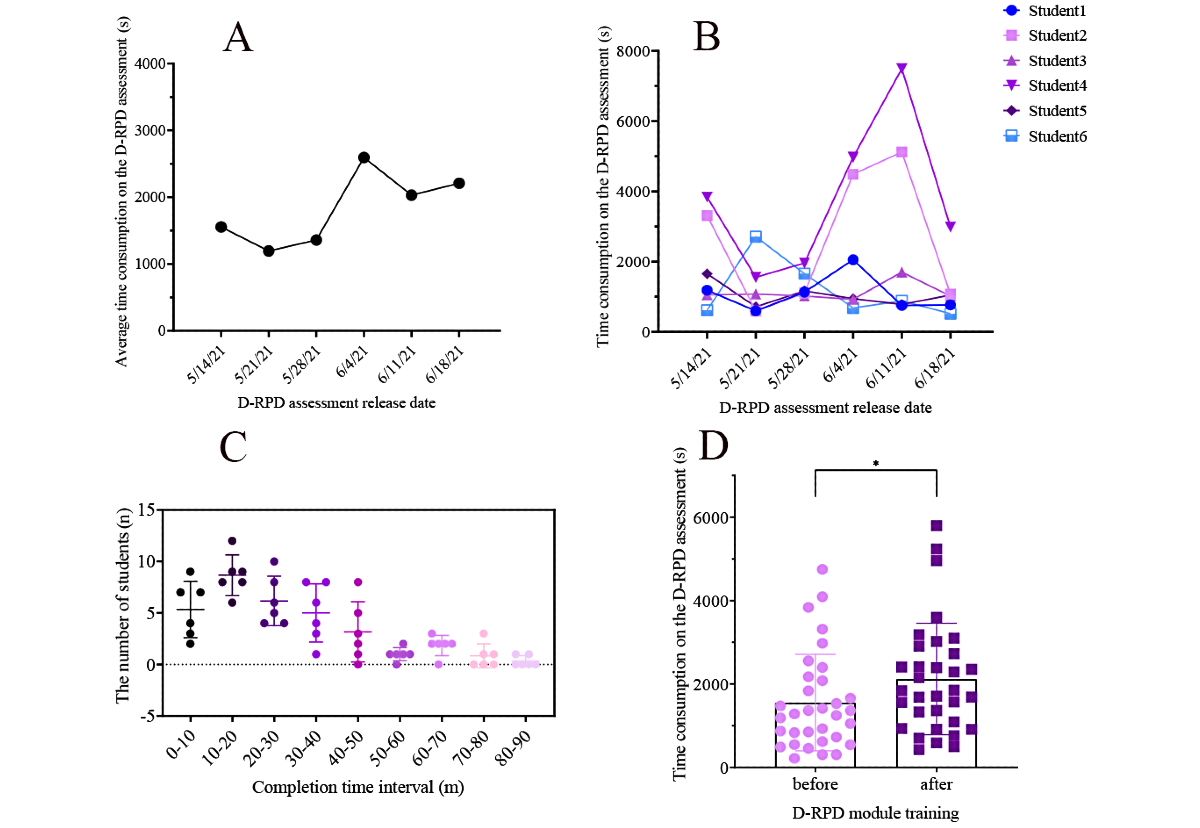
Experimental results of the removable partial denture design (time dimension). A: Average time consumption of the removable partial denture design assessment (33 students in 6 weeks); B: Time consumption of the removable partial denture design assessment of a sample team (6 students in 6 weeks); C: Distribution of the removable partial denture design assessment completion (33 students in 6 weeks, mean [SD]); D: Paired Mann-Whitney analysis results of the time-dimension data set (*P*=.04). D-RPD: removable partial denture design.

According to the design of performance evaluation, 33 students completed the 7-week practice task. Three students withdrew from the experiment for personal reasons; therefore, their scores were excluded from the statistical analysis. Owing to a system error in the D-Manager module in the first week, the task completion time data in the first week failed to be collected. The resulting complete data set included the scores of the 33 students who had practiced D-RPD for 7 consecutive weeks, as well as task completion times for the next 6 weeks. Paired *t* test analysis was performed on the score-dimension data set, with the mean scores of the first 2 weeks as the pretest and the mean scores of the last 2 weeks as the posttest. Since the time consumption was not normally distributed, the time consumption data of the second week at the beginning of the test were used as the pretest, and the average time consumption data of the last 2 weeks were used as the posttest. Mann-Whitney analysis was performed on the time-dimension data set. The results of the complete data set analyses are shown in Figure 10 and Figure 11.

The results showed significant differences and fluctuations in the average scores. For the first 4 weeks, it was 85.65 out of 100. For the fifth week, it fell slightly to 79.60. At this stage, students reported some irritability such as feeling more stressed and less novelty in web-based work and more tough training exercises in the last 3 weeks. However, this finding was expected at the beginning of the design. Students’ scores rebounded after they were given proper rest and educator incentives to continue with the exercise program. For the last 2 weeks, the average score rose above 91.15. The time consumption curve was highly complementary to the score curve. In the trough of the score curve, the time consumption curve reached its peak. The data of the 2 dimensions provide cross-evidence for educators to observe students’ learning progress. At the same time, the data set supports hierarchical real-time observations from the individual to group to class dimensions and enables educators to identify areas of time consumption that were centrally distributed among the testers. Most participants in this experiment took 30-40 minutes to complete 1 D-RPD assessment. Statistical analysis of the score-dimension data set showed that students’ D-RPD scores increased significantly after the D-RPD exercise (*P*=.002). The results of the statistical analysis of the time-dimension data set showed that the time consumed by the students on a single task increased significantly after the D-RPD exercise (*P*=.04).

#### Phase II

According to the design of performance evaluation, 28 of 30 students (93% response rate) completed the questionnaire and their responses were determined to be valid. In this study, Cronbach α was .980, suggesting that the reliability and the internal consistency of the statistics were sufficient. Obviously, all indicators exceeded the pass mark, proving the effectiveness of the system. Table 1 shows the mean (SD) score on each item.

In the open-ended questions, some students reported that systems were sometimes difficult to connect. However, investigations revealed that these problems were mainly due to errors, as users did not clear their browser cache in time. Other students reported the effectiveness of instructor supervision in the use of the system and felt that instructor supervision facilitated their completion of practice tasks. Moreover, mainly students with better practical skills but poorer performance in theory exams approved the system’s facilitation of skills exams because they felt that OMEDT and the OMEDT system could more fairly evaluate their abilities.

**Table 1:** Feedback from long-term users of the Objective Manipulative Skill Examination of Dental Technicians system.

Item	Mean (SD)	Skewness
The OMEDT^a^ system helps me to learn more efficiently.	4.14 (0.990)	–1.479
The OMEDT system can help me to be more efficient in my future work.	4.10 (1.047)	–1.423
The OMEDT system has improved my theoretical knowledge.	3.79 (1.177)	–0.982
The OMEDT system has improved my practical skills.	4.00 (1.035)	–1.038
I was able to use the OMEDT system to study independently without being asked to do so by the instructor.	4.14 (1.060)	–1.840
I may use the system to review in the future in case of interim work or exams.	4.10 (0.939)	–1.607
The OMEDT system is beneficial for dental technicians.	4.14 (0.990)	–1.479
I am willing to take the practical skills exam using the OMEDT system.	4.10 (1.047)	–1.423

^a^OMEDT: Objective Manipulative Skill Examination of Dental Technicians.

## Discussion

### Principal Findings

Dental technology is characterized as an interface between disciplinary knowledge and the field of professional practice. The teaching, learning, and assessment of dental technology should facilitate connecting discipline-specific theory to laboratory-based practice to the design and fabrication of prostheses. The professional skills necessary to perform such work must be rigorously assessed and periodically examined after certification is obtained to ensure that the practitioner’s performance level is always able to meet the needs of the industry. This is the essence of the CDT practical examination. Unfortunately, conducting the CDT practical examination is a global problem. The United States does not have a national certification system for dental technicians. The title of CDT is necessary for educating prospective CDTs and to manage a certified dental laboratory. This is obtained by passing the CDT examination, including written and practical examinations, offered by the National Board for Certification in Dental Laboratory Technology established by the National Association of Dental Laboratories [46]. The United Kingdom has certifications for dental technicians and clinical dental technicians but does not have the CDT examination [47]. New Zealand has a certification, including a written examination for dental technicians, which is obtained by passing the New Zealand Dental Technology Registration Examination offered by the Dental Council [48]. In China, to qualify as a CDT, one must pass the CDT examination provided by the Ministry of Health and Welfare [40]. As in the United Kingdom, this is a written examination only. It is challenging to organize a real material-based examination for test purposes. For example, the whole casting process of metal crown fabrication usually needs more than 12 hours. Owing to the long preparation time and expensive material consumption, it is difficult for the CDT practical examination institutions to prepare semifinished casting products for each candidate. The examination results largely depend not only on the professional performance of the candidates but also on the quality of those semifinished casting products. Considering the potential for fraud and ethical issues, it is unacceptable to examine the final product’s quality only or send the material package and require candidates to prepare semifinished products themselves for the CDT practical examination (which is the American plan) [46].

Some possible solutions based on virtual simulation technology have been applied in dental education. Liu et al [49,50] reported that the application of VLNP and RDTES demonstrated some promising features for students’ training in preclinical operative and ceramic crown preparation. Osnes et al [51] reported applying a haptic device (Simodont, Nissin Dental Products Europe BV) in the prosthodontics and endodontics areas separately. Different studies [52,53] have shown that Simodont significantly improves students’ psychomotor skill performance in these areas. Studies [54-56] have shown the application of a haptic device named Virteasy Dental, and the training of psychomotor skills by using Virteasy Dental can be extended to the fields of implantology and endodontics. Considering the association between dental technology and the stomatology specialty, the performance of virtual simulation technology in dental technology education can be optimistically predicted.

The limitation of the existing virtual simulation products is that most of them are aimed at a single discipline in stomatology. Haptic devices and VLNP and RDTES are separate and lack integration. Moreover, there are no well-known educational or examination standards for dental simulators or associated exercises [20,39,57]. When virtual simulation technology was attempted for the CDT practical examination, existing products were unfortunately not targeted. The OMEDT system, as the world’s first virtual simulation system based on professional requests for dental technology studies, can fill the gap of virtual simulation education in dental technology. Virtual simulation technology limits the length of time in a single examination station, and the V-H-R architecture ensures the diversity in the examination content. The examination mode based on OSCE ensures that students’ professional skills are assessed completely. The effectiveness of OMEDT and the OMEDT system was verified by student evaluations. Most of the students gave positive comments on the usefulness of the OMEDT system for their studies and examinations and believed that the OMEDT system helped them evaluate their professional ability more fairly and comprehensively. Further, students expressed a strong desire for self-directed learning and continuing education during work, demonstrating that the OMEDT system can be used as part of the CDT practical examination.

The V-H-R architecture (1 program module) of OMEDT can be used for multiple curricula. For example, D-RPD can be used as a tool by educators to demonstrate and explain the Kennedy Classification during theory lectures on removable denture technology. D-RPD can also be used as a tool by educators to present and explain the design of RPDs or by students to complete the design task of the RPD during their experimental classes on RPD technology. Further, D-RPD can be used as a tool by students during their internship and graduation to practice freely and as a tool for instituting graduation exams. The OMEDT system can be used by students from their first exposure to professional knowledge and continue to be used in the final vocational examination, which could help unify teaching objectives and examination standards. Thus, the OMEDT system is an integrated solution for the CDT practical examination. China may eventually be able to institute a formal CDT practical examination based on the OMEDT system.

Our study also extends the concept and application of RDTES technology. Traditionally, RDTES devices are often described as 2D or 3D image acquisition devices [58] that record and store the working process of students by using a simulator [59]. Whether this technology actually improves dental students’ cognitive ability and psychomotor skills is still as matter of debate. In the field of dental technology education, the practical significance of this technology is weakened, because most of the operations involved in the dental laboratory are not independent, but rather need as many as a dozen processes—a total time of approximately 5-7 days is required. This is part of the reason that the dental virtual simulator cannot be directly used in dental technology, as mentioned in the previous discussion. Considering the characteristics of dental technology specialties, it is important to evaluate and observe students in real time from the knowledge of the results [58] dimension and reconstruct the concept and logic of RDTES technology in the field of dental technology. According to “A scoping review of simulation-based dental education” by Dr Higgins [60], the training of aspiring dentists by using simulation-based technology can be done in phases. These phases include briefing, simulation, feedback, debriefing, reflection, and evaluation. Diversity in students’ learning styles and motivation is the crucial challenge that course designers face. The RDTES part of the OMEDT system draws on and develops this teaching theory. In addition to the traditional RDTES design, the application of the OMEDT system in teaching practice provides a new evaluation idea for the process evaluation of skills. In conventional dental education, especially in the cultivation of skills, educators are more inclined to evaluate students’ skills based on the results. The limitations and inaccuracies of this evaluation standard are apparent. The OMEDT system provides educators with the perspective of growth curve observation, carries out the process assessment of students’ psychomotor skills, and can be predicted to be more helpful for cultivating students’ psychomotor skills. Educators can more easily make timely adjustments and provide prompt responses during long large-scale training sessions. This will be a new dimension of evaluation of the knowledge of results. This can be illustrated briefly by the application of D-RPD. In the general pedagogical experience, the knowledge point of D-RPD has been set in the concept that 3 class exercises were enough. However, the growth curve analysis provided by the OMEDT system led to other conclusions. It takes more than 5 rounds of continuous design training to show the training effect, which suggests that educators may need to adjust the setting of courses or change the routine habit of assessments. Meanwhile, the observation perspective based on the time dimension of D-Manager helps educators pay timely attention to students’ learning motivation and feedback in the continuous task. For example, in the fifth week, the completion time increased significantly. At the same time, the average score decreased, suggesting that educators should pay attention to and adjust the task’s difficulty or find the possible problems in students’ learning. The time dimension also references the formulation of OMEDT’s section time. For example, the validity evaluation experiment of D-RPD suggests that most students can complete a D-RPD within 30-40 minutes, which is reflected in the architectural design of the OMEDT station (D-RPD station is set at 45 minutes). The introduction of the OMEDT system in the dental technology curriculum and the utilization of its data to stratify dental technology students and predict their psychomotor skill performance provide the opportunity to tailor the learning process to meet the individual diversity in students’ expertise and allow students to work at their own pace. In this context, the major core curriculum of dental technology could provide an education that leads to the optimal performance of each student. Computer engineers play an indispensable role in the development of new systems. In the process of the experiments, the engineers helped to maintain the system during running time and to collect training data. In addition, the engineers could help students develop good habits of virtual simulation technology application (such as timely cleaning of browser cache), so that students can use the system to study and test more smoothly.

### Limitations and Future Work

The main limitation of this study is that owing to time limitations, a comprehensive effectiveness evaluation was not performed for all modules of the OMEDT system. This limitation will be addressed in subsequent studies. In addition, some modules of the OMEDT system were found to have room for improvement in teaching practice. These modules will be improved in the subsequent research plan. OMEDT and the OMEDT system mainly examine the cognitive ability and psychomotor skills in professional performance but not the emotional skills of the dental technicians. The empathy and communication ability of dentists and dental technicians are important factors that determine the success rate of oral clinical treatment. OMEDT and the OMEDT system should continue to develop and explore the scope of this framework in the field of dental interprofessional education. In the future, the development of a professional performance training system for students with stomatology and dental technology majors should be further discussed. The practical application of interprofessional education in dental health education will become the research frontier in the future.

### Conclusion

As the world’s first virtual simulation system for dental technology education, the OMEDT system integrates 3 existing virtual simulation technologies, that is, VLNP, a haptic interaction, and RDTES. In a virtual environment that saves cost, resources, and manpower, the OMEDT system provides students with a “virtual laboratory” for repeated independent practice. Large-scale data collection provides and validates new dimensions for educators to observe students’ professional performance in experimental teaching activities, which is of positive significance to the assessment of students’ learning process. The OMEDT system has positive potential as an overall solution to the CDT practical examination. In the future, we will improve OMEDT according to the collected data and conduct more experiments to evaluate the training effectiveness of other modules in the OMEDT system.

## Abbreviations

CDT: certified dental technician
DOF: degrees of freedom
D-RPD: removable partial denture design
OMEDT: Objective Manipulative Skill Examination of Dental Technicians
OSCE: Objective Structured Clinical Examination
RDTES: real-time dental training and evaluation system
RPD: removable partial denture
VLNP: virtual learning network platform
